# Supplementary material for: ISCA2 inhibition decreases HIF and induces ferroptosis in clear cell renal carcinoma
Source: Oncogene. 2022 Sep 12;41(42):4709–23. doi: 10.1038/s41388-022-02460-1 (PMC9568429; doi:10.1038/s41388-022-02460-1)
Supplement: Supplementary file 1 — Supplemental Figures [file 41388_2022_2460_MOESM1_ESM.pdf]

A)

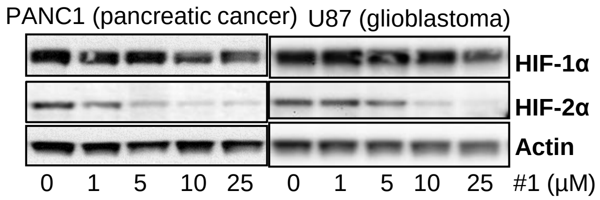

B)

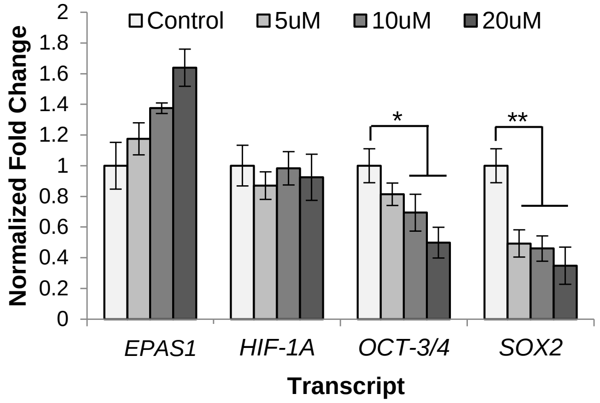

C)

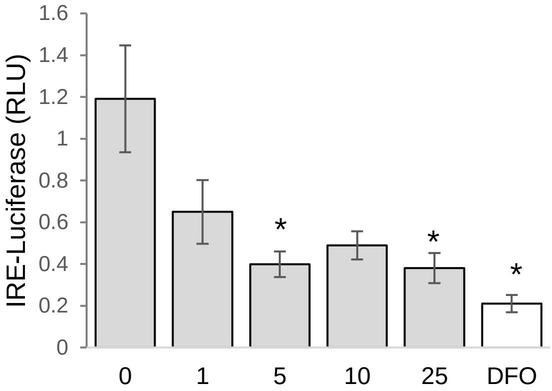

D)

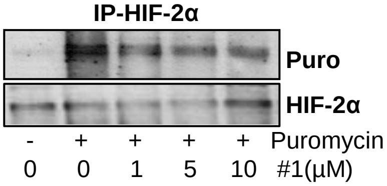

Supplemental Fig 1: A) Western blots showing the effects of 24 hours' treatment of PANC1 and U87 cells with indicated concentrations of compound #1. Cells were exposed to hypoxia (1%O_2) for 24 hours to induce HIF expression. B) Quantitative RT-PCR showing effects of #1 on the transcription of *HIF1A, HIF2A* (*EPAS1*) and HIF-2 target genes *OCT-3/4* (*POU5F1*) and *SOX2* in hypoxic ACHN cells. Effect of compound #1 on luciferase activity driven by a HIF-2α Iron-Responsive Element (IRE)-luciferase reporter construct transiently transfected into ACHN cells. * $p < 0.05$. D) Western blot showing puromycin incorporation into immunoprecipitated HIF-2α in hypoxic ACHN cells treated with #1 for 24 hours.

Supplemental Figure 2

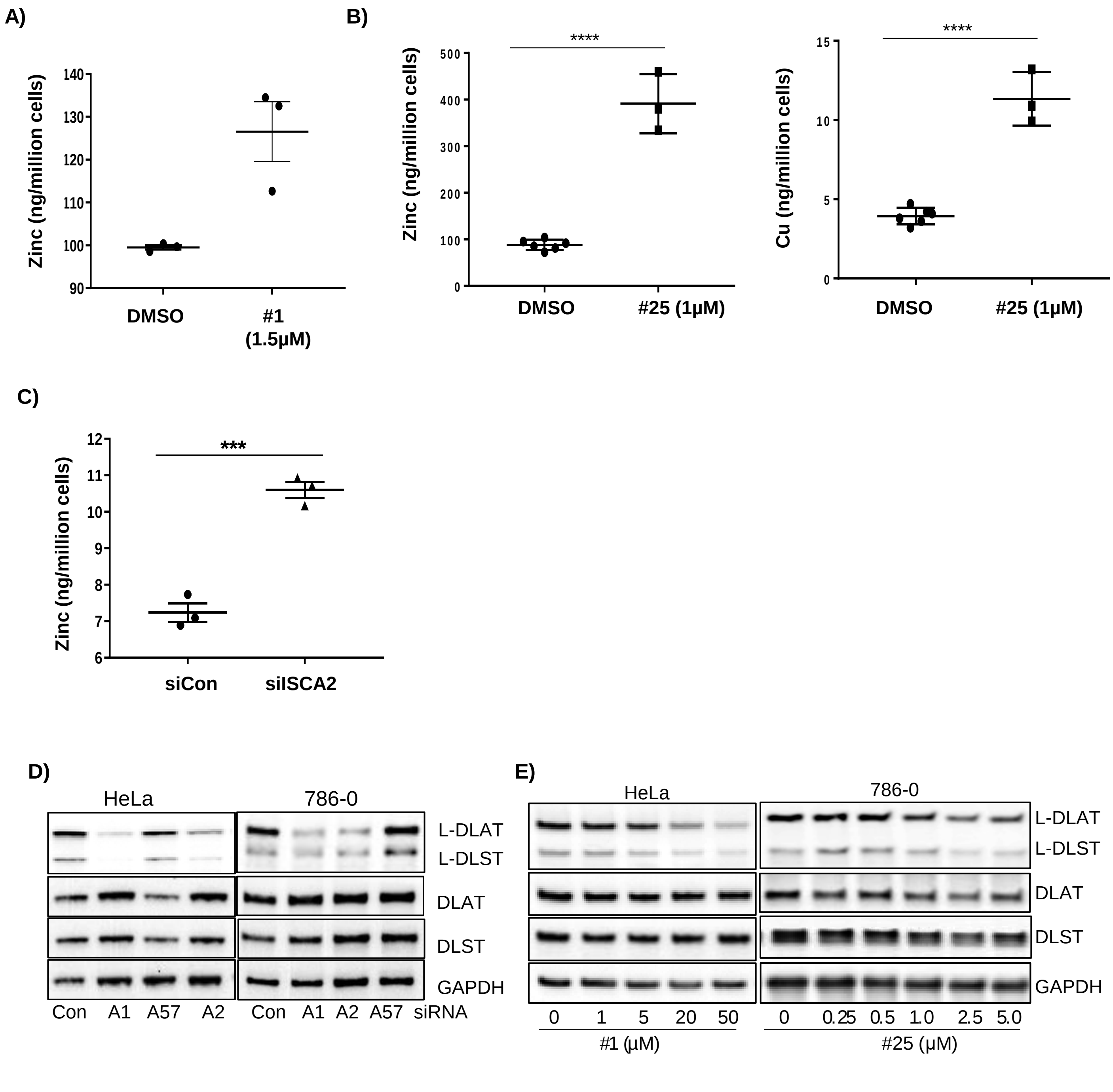

Supplemental Fig 2: Metals content of 786-0 cells treated with; A) #1 (24 hours), B) #25 (24 hours) or C) Control or ISCA2 siRNA (6 days) as determined using ICP-MS. D) Western blots showing effect of indicated siRNA transfections (6 days) on lipoylation of DLAT and DLST in HeLa and 786-0 cells. E) Effect of #1 or #25 treatment (24 hours) on lipoylation of DLAT and DLST in HeLa and 786-0 cells, respectively.

Supplemental Figure 3

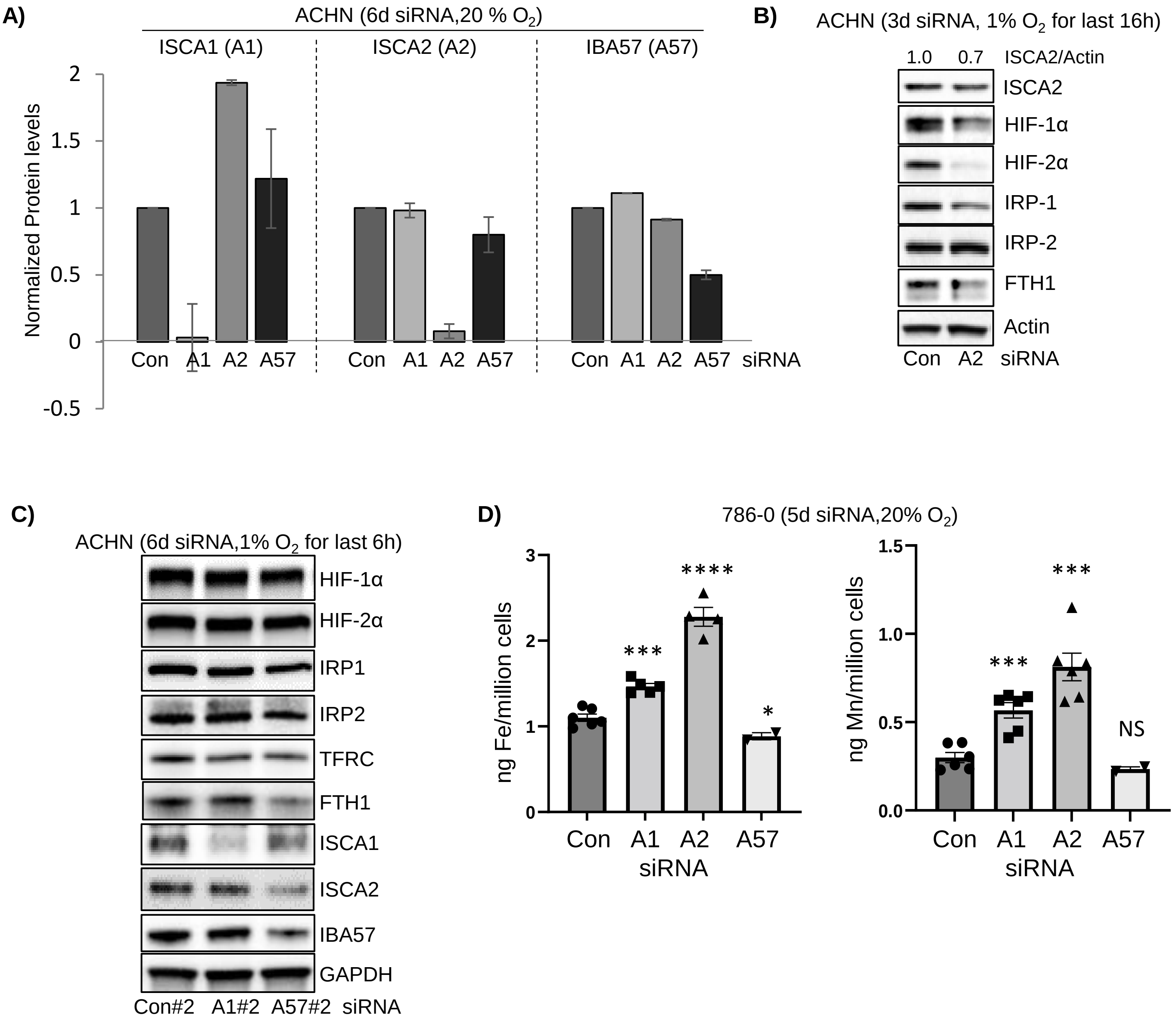

Supplemental Fig 3: A) Effect of 6-day transfection with non-targeting siRNA (Con), ISCA1 (A1), ISCA2 (A2) or IBA57 (A57) on levels of their respective proteins by densitometry of their respective bands normalized to GAPDH in ACHN cells. Results are average of 2 independent experiments ±SD. B) Effect of 3-day transfection of ISCA2 siRNA (A2) on levels of indicated proteins in hypoxic ACHN cells. Densitometric values of ISCA2 normalized to GAPDH are shown above blots. C) Effect of 6-day knockdown of ISCA1 and IBA57 on indicated proteins in hypoxic ACHN cells. D) Effect of 5-day siRNA knockdowns on metals accumulation in 786-0 cells detected using ICP-MS. Approximately equal cell numbers were submitted for analysis. All data are representative or averages of at least two independent experiments ±SEM.

Supplemental Figure 4

siCon

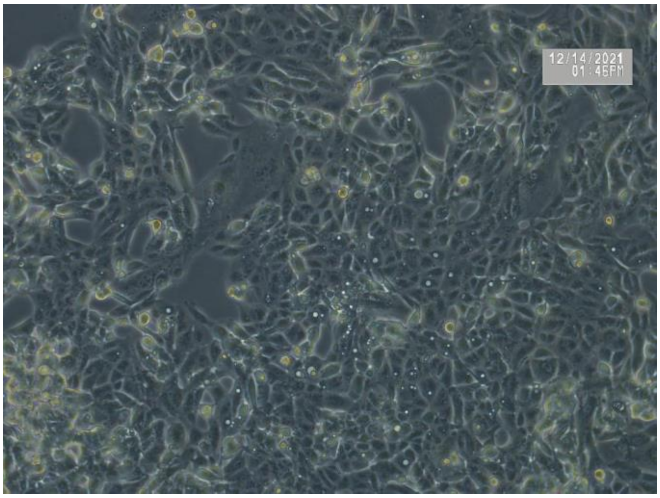

siISCA1

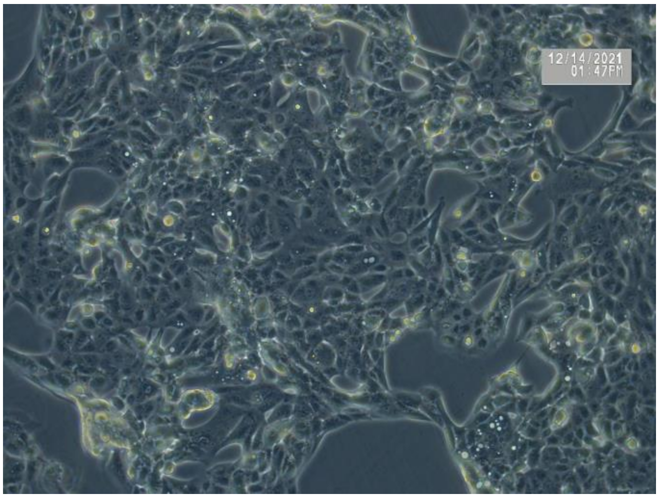

siISCA2#1 (OTP-02)

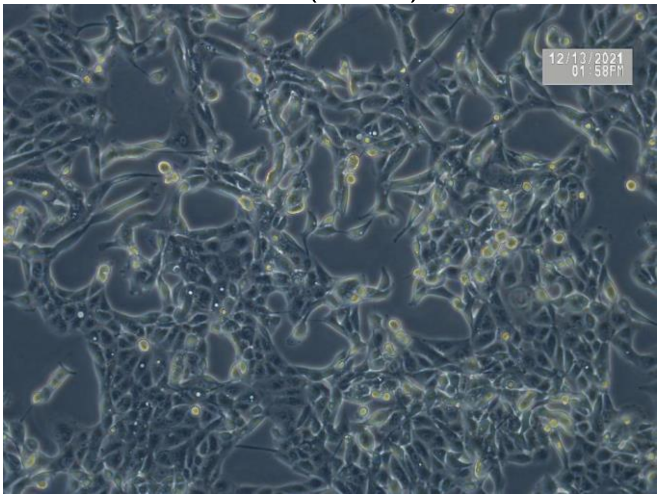

siISCA2#2 (siGenome – 01)

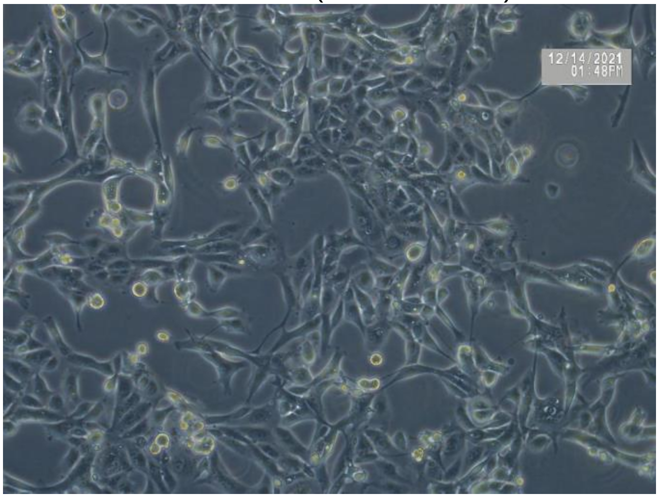

siIBA57

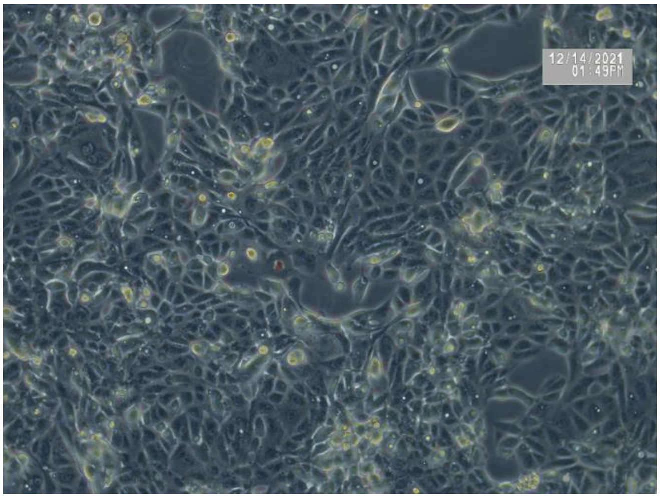

Supplemental Fig 4: Representative phase contrast photomicrograph of adherent ACHN cells in flasks after 5 days transfection with indicated siRNAs used for ICP-MS analysis.

siCon

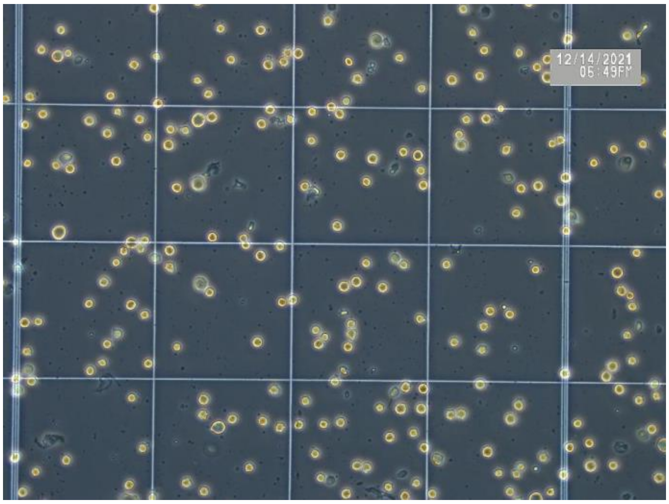

siISCA1

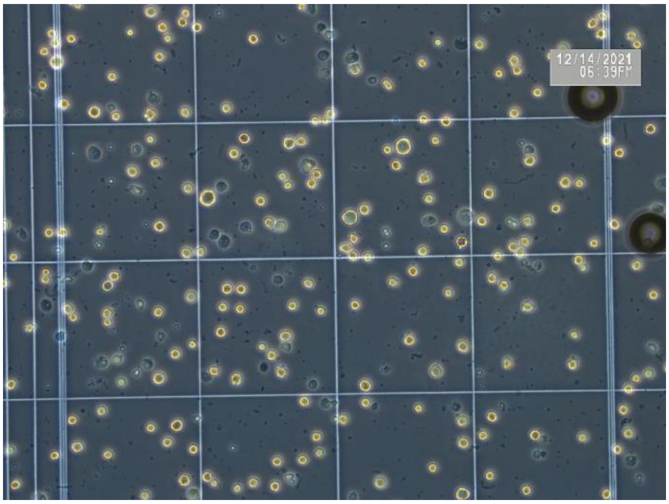

sISCA2

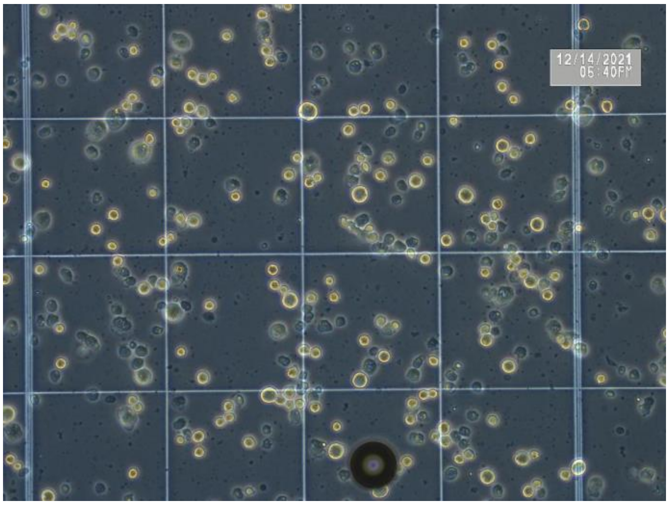

siIBA57

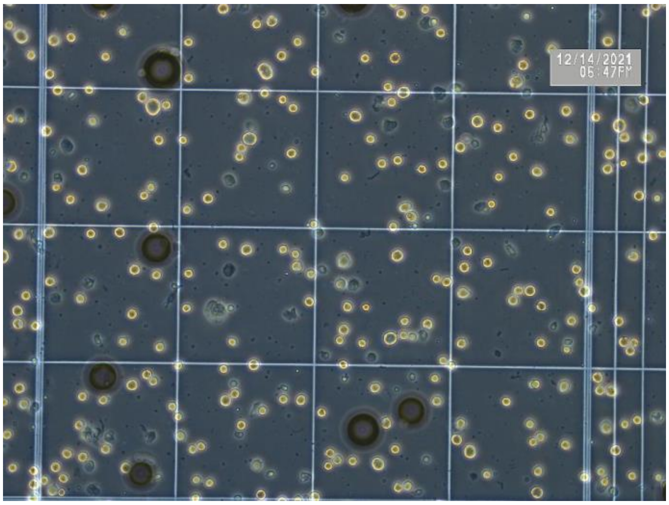

Supplemental Fig 5: Representative phase contrast photomicrographs within hemocytometer counting chambers of trypsinized ACHN cells after 5 days transfection with indicated siRNAs used for ICP-MS analysis. Images indicate the increased size and reduced number of cells transfected with siISCA2

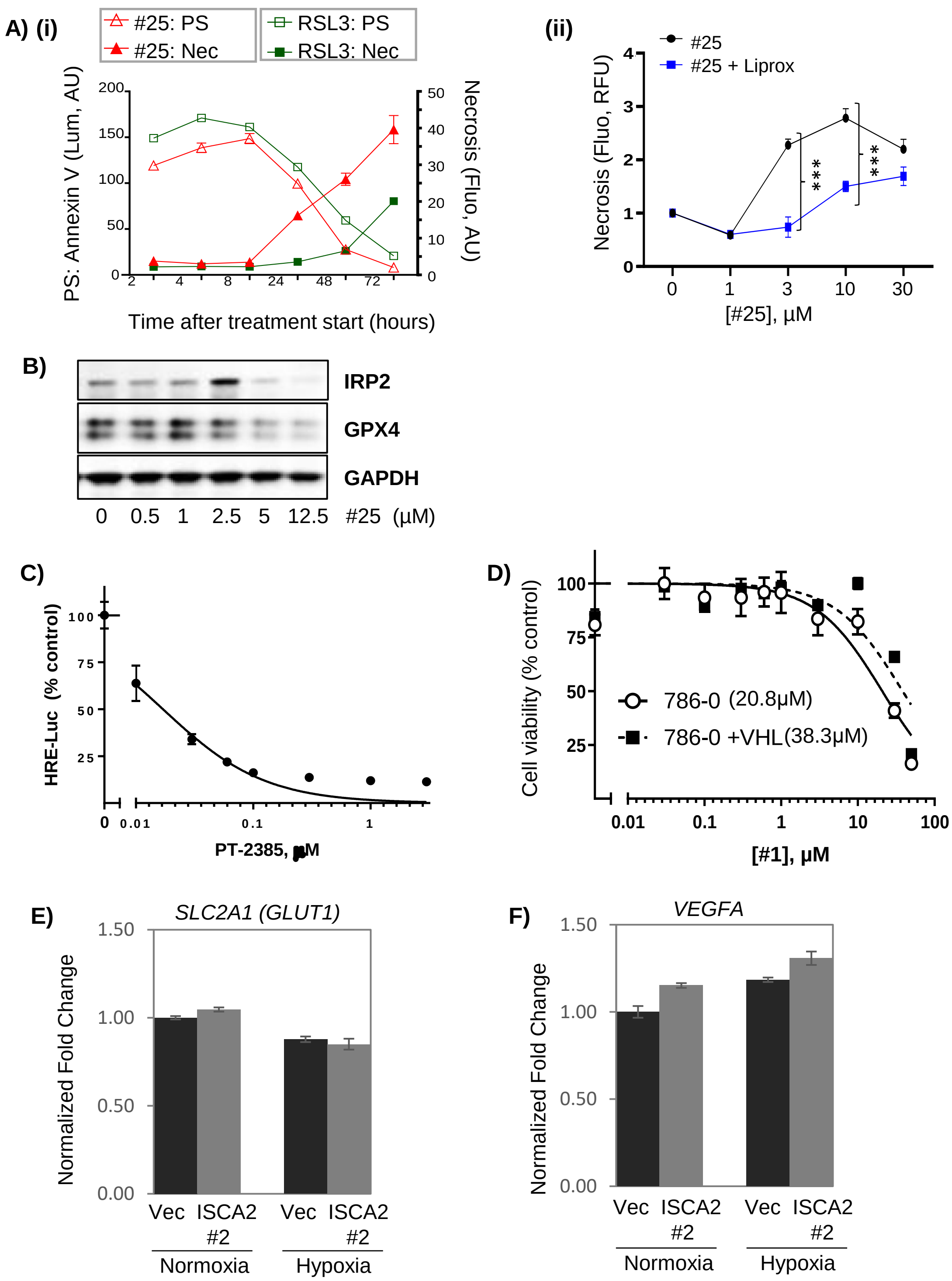

Supplemental Fig 6: A) (i) Time course of PS: annexin V binding (left Y-axis) and loss of membrane permeability (i.e. necrosis - right Y-axis) in RCC10 cells after #25 (3µM) or RSL3 (0.5µM) treatment. Data shown are the mean ± SD; (ii) Dose response effect of #25 ± liproxstatin (1µM) on inducing loss of membrane permeability (necrosis) after 72hrs' treatment. B) Western blot showing effects of treatment with #25 on GPX4 and IRP2 at higher concentrations. Blots show reductions in IRP2 and GPX4 at ≥5µM treatment. C) 786-0 HRE-Luciferase dose-response assay of PT2385 (24hr). D) Resazurin cell viability assay of 786-0 parental or 786-0 cells with pVHL reconstitution treated with #1 for 72 hours. Average IC_{50} values (µM) are shown in brackets.E,F) Normalized gene expression levels (via qRT-PCR) of SLC2A1 and VEGFA and in RCC10 vector or ISCA2 overexpressing cells in normoxia or hypoxia (24h). Data were determined using ΔΔCt method normalized to β2-microglubulin.

A)

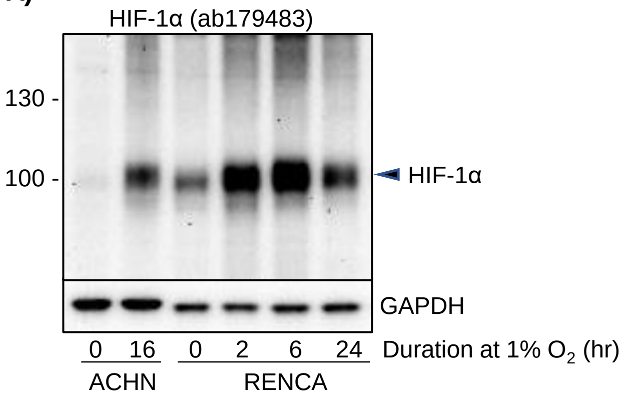

B)

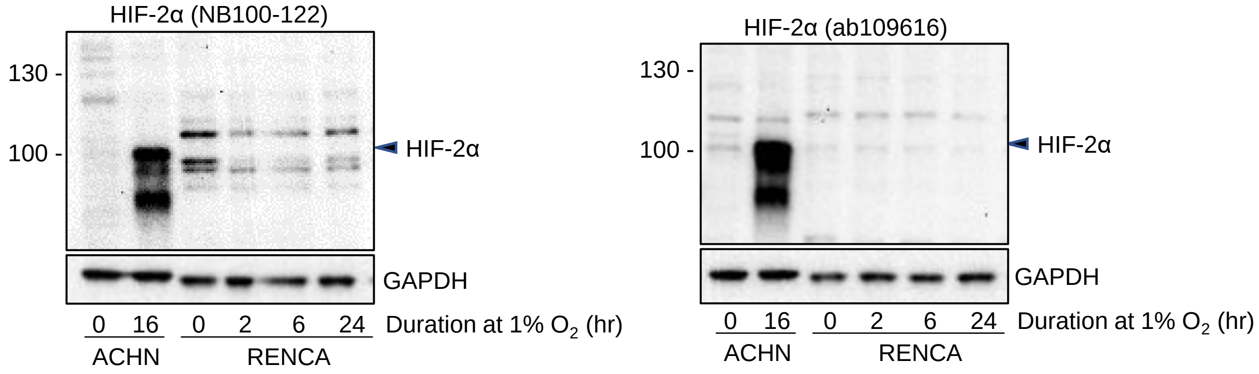

Supplemental Fig 7: Western blots validating HIF-1α and lack of HIF-2α expression in RENCA cells. ACHN (human renal cancer cells) and RENCA cells (mouse renal adenocarcinoma cells) were grown in normoxia (20% O_2) or exposed to hypoxia (1% O_2) for indicated durations. Cells were lysed and probed with antibodies designed to recognize both human and mouse forms (according to the manufacturer) of HIF-1α (A) or HIF-2α (B). Arrows indicate expected molecular weights for HIF-1α and HIF-2α.
